# MicroRNA-21 promotes hepatocellular carcinoma HepG2 cell proliferation through repression of mitogen-activated protein kinase-kinase 3

**DOI:** 10.1186/1471-2407-13-469

**Published:** 2013-10-10

**Authors:** Guangxian Xu, Yilin Zhang, Jun Wei, Wei Jia, Zhaohui Ge, Zhaobo Zhang, Xiaoming Liu

**Affiliations:** 1 General Hospital of Ningxia Medical University, Yinchuan 750004, China; 2 School of Laboratory Medicine, Ningxia Medical University, Yinchuan 750004, China; 3 Huashan Hospital of Fudan Medical University, Shanghai 20040, China; 4 College of Life Science, Ningxia University, Yinchuan 750021, China

**Keywords:** miR-21, Hepatocellular carcinoma, MAP2K3, HepG2, miRNA sponges

## Abstract

**Background:**

microRNA 21 (miR-21) has been demonstrated to be significantly elevated in many types of cancers, including the hepatocellular carcinoma (HCC). In the present study, we investigated the role of miR-21 in HCC by identifying its novel targets, as well as its underlying molecular mechanism.

**Methods:**

The expression of mitogen-activated protein kinase-kinase 3 (MAP2K3) in human HCC tumor tissues and adjacent non-tumor tissues was determined by immunohistochemistry staining (IHC) analysis. The 3’-untranslated region (3’-UTR) of MAP2K3 combined with miR-21 was experimentally verified by a miRNA luciferase reporter approach. Moreover, the role of miR-21 in regulating HCC cell proliferation was analyzed by an MTT assay infected with miR-21mimics/sponge inhibitor Adenoviral viral vectors.

**Results:**

By immunohistochemistry staining analysis, we found that mitogen-activated protein kinase-kinase 3 (MAP2K3) was strikingly repressed in the human HCC tumor tissues, in comparison with the adjacent non-tumor tissues in clinical settings. More importantly, the repression of MAP2K3 was inversely correlated with the expression of miR-21 in HCC. Further study demonstrated that the MAP2K3 was a novel direct target of miR-21, which was experimentally validated by a miRNA luciferase reporter approach. In HepG2 cells, inhibition of miR-21 expression with an adenoviral miR-21 sponge vector profoundly suppressed cell proliferation by up-regulating MAP2K3 expression at both mRNA and protein levels.

**Conclusions:**

These results provide a clinical evidence that MAP2K3 may be a tumor repressor gene, and it is a direct target of miR-21 in HCC, indicating an underlying mechanism by which miR-21 is able to directly target MAP2K3 and inhibit its expression during the carcinogenesis of HCC, at both transcriptional and post-translational levels. This study also suggests that targeting miR-21-MAP2K3 pathway may be a promising strategy in the prevention and treatment of HCC.

## Background

MicroRNAs (miRNAs) are evolutionarily conserved, endogenous, single-stranded, non-coding RNA molecules with a fundamental role in the regulation of gene expression [1]. miRNA binds a target gene through imperfect basepairing to the complementary sequences in the 3’untranslated region (3’UTR) of a gene to transcriptionally or post-transcriptionally suppress its expression at the mRNA or protein levels in many organisms, such as yeast, fruit flies, worms, vertebrates, human and plants [2]. An increasing number of studies has uncovered that the expression of miRNAs is deregulated in many types of cancers in comparison with matched non-neoplastic tissues, including the hepatocellular carcinoma (HCC) [3]. Among them, miR-21 is aberrantly expressed in almost all epithelial cell-derived solid tumors including breast, pancreas, lung, stomach, prostate, colon, head and neck, liver, and esophageal cancers [4], as well as in hematological malignancies such as leukemia, lymphoma and multiple myeloma [5,6]. Further analysis of the tumors implicated a variety of signaling pathways in which the miR-21 plays a pivotal role in the carcinogenesis of many types of cancers [7-11], and some of the signaling molecules have been experimentally validated as targets of miR-21, including phosphatase and tensin homolog (PTEN) [10,12], programmed cell death 4 (PDCD4) [13], reversion-inducing -cysteine-rich protein with kazal motif (RECK) [14], and tropomyosin alpha-1 chain (TPM1) [11].

Owing to the increase of hepatitis B virus (HBV) and hepatitis C virus (HCV) infection, aflatoxin-contaminated food, and alcohol abuse, the incidence of hepatocellular carcinoma (HCC) is constantly rising in the last two decades, particularly in China, where the HCC is one of the most frequently occurring cancers [15]. Genetic and expression profiling studies of HCC have demonstrated the alterations of a variety of mutations and expression of oncogenes and/or tumor-suppressor genes in the carcinogenesis of liver cancer, which are often concomitant with the deregulation of an important signaling pathway, such as p53, AP-1, and the mitogen-activated protein kinases (MAPKs) pathway [16,17].

The MAPKs generally expressed in all cell types functionally to transduce extracellular signals into various intracellular responses [18]. In addition, dysregulation of MAPK signaling pathway was often found in various types of cancers, including the HCC, which was also phenotypically validated in genetic mouse models with impaired MAPK signaling [16,19]. Previous studies have revealed that the expression of miR-21 was augmented in malignant HCC tissues relative to the benign HCC and normal liver tissues [20], however, its underlying regulatory mechanism has not been fully elucidated yet. Recently, Jia et al. found that mitogen-activated protein kinase-kinase 3 (MAP2K3) was remarkably down-regulated in breast cancer epithelial cells [21], we therefore hypothesize that miR-21 may play a role in regulation of MAP2K3 in HCC pathogenesis. In this study, we found that the mitogen-activated protein kinase-kinase 3 (MAP2K3) was markedly down-regulated in human HCC tissue, compared with adjacent non-tumor tissues for the first time. The MAP2K3 was further identified and experimentally validated as a novel target for miR-21. This study might provide a new avenue for comprehensively understanding the regulatory mechanism of miR-21 in cancers in general, and the HCC in particular.

## Methods

### Ethics statement

Human liver tissue was collected with a protocol approved by the Ethic Committee for the Conduct of Human Research at Ningxia Medical University. Written consent was obtained from every individual according to the Ethic Committee for the Conduct of Human Research protocol. All participants were over 18 years of age and provided written informed consent for the publication of the data. This study was approved by the Ethic Committee for the Conduct of Human Research at Ningxia Medical University.

### Human liver tumor samples

Fourteen liver tumor samples with histologic evidence of HCC, and matched adjacent non-tumor tissues without histological evidence of HCC were archival samples from department of Medical Pathology Department, General Hospital of Ningxia Medical University from 2007 to 2009 (Table 1) [22].

**Table 1:** The expression of MAP2K3 in human HCC tissues determined by IHC

**Sample ID**	**Age**	**Gender**	**Tumor size (cm)**	**Tissues**	**IHC staining**	
					**IA value**	**Scores**
1	56	Male	3.5	Tumor	295.26	-
				Non-tumor	5324.96	+++
2	69	Male	7.4	Tumor	81.52	-
				Non-tumor	868.44	+
3	27	Female	5.8	Tumor	697.8	-
				Non-tumor	1509.85	+
4	42	Male	5.3	Tumor	237.82	-
				Non-tumor	6341.06	+++
5	62	Female	4.4	Tumor	387.59	-
				Non-tumor	7880.92	+++
6	33	Female	1.5	Tumor	145.52	-
				Non-tumor	1554.87	+
7	52	Female	2.7	Tumor	4456.78	++
				Non-tumor	12345.41	+++
8	40	Male	6.2	Tumor	750.19	+
				Non-tumor	4249.48	++
9	47	Male	5.7	Tumor	208.14	-
				Non-tumor	1824.86	+
10	72	Male	3.3	Tumor	234.06	-
				Non-tumor	752.39	+
11	65	Male	4.0	Tumor	117.6	-
				Non-tumor	6744.49	+++
12	51	Female	5.0	Tumor	519.31	-
				Non-tumor	2628.37	+
13	60	Male	7.0	Tumor	1552	+
				Non-tumor	566.92	+
14	30	Male	2.2	Tumor	1301.41	+
				Non-tumor	15692.81	+++

### Cell culture and transfection

Cell lines of human embryonic kidney 293 and human hepatoma cell HepG2 were purchased from American Type Culture Collection (Mannasas, VA, USA). The cells were cultured and maintained at 37°C in a humidified atmosphere of 5% CO_2_ 95% air in dulbecco’s modified eagle medium (DMEM) supplemented with 10% Fetal Bovine Serum (FBS) and 1% pen/strep. Plasmid DNA transfection was performed using TransLipid Transfection Reagent (Beijing TransGen Biotech Co. Ltd, Beijing, China) per manufacturer’s instruction.

### Generation of recombinant adenoviral vectors

In order to generate adenoviral vectors overexpressing miR-21, oligonucleotides of miR-21 forward (5’-TAGG*GGTACC*CCTAAACCAACCAGCCAACC-3’) and reverse primer (5’- TATGC*TCTA GA*GCTCCGGCTTTAACAGGTG -3’) were synthesized, and respective restriction sites of *Kpn* I and *Xba* I were introduced at 5’-ends, based on the sequence of human miR-21 (5’- uagcuuaucagacugauguuga-3’, MIMAT0000077) from miRBase database. Similarly, in order to produce a miR-21 sponge vector, annealed double strands containing 8× tandem of binding sites that are perfectly complementary to miR-21 seeding sequence, was generated [23]. The sense sequence of miR-21 sponges with a Kpn I and a Hind III sites at ends was listed below: 5’-ATTC*GGTACC*TAGCTTATGTCCTGATGTTGATAGCTTATGTCCTGATGTTGATAGCTTATGTCCTGATGTTGATAGCTTATGTCCTGATGTTGATAGCTTATGTCCTGATGTTGATAGCTTATGTCCTGATGTTGATAGCTTATGTCCTGATGTTGATAGCTTATGTCCTGATGTTGATCTAGA*AAGCTT*GGCG-3’. The double stranded oligonucleotides were further modified with appropriate restricted endonucleases and cloned into an adenoviral shuttle vector, pAdTrack-CMV (Department of Biological Chemistry, School of Medicine, Fudan University, Shanghai, China). The resulted proviral shuttle plasmids were used for generation adenoviral vector expressing miR-21 and miR-21 sponge following a protocol described previously [24,25]. The adenoviral vectors were designated as Ad/pri-miR-21 for expressing miR-21, and Ad/miR-21/inhibitor for expressing miR-21 sponge in this study. A control empty adenoviral vector, Ad/con was also generated. The viral functional titration was essentially performed using Spearman-karber method as described in the previous study [26].

### Infection of HepG2 cells with the adenovirus

The HepG2 cells were seeded in 6-well tissue plate and grown to 80–90% confluence prior to infection. Cells were infected with Ad/pri-miR-21, Ad/miR-21/inhibitor or Ad/con at a multiplicity of infection (MOI) of 10, and the cells were continued to culture for additional 24 h before they were harvested for analysis.

### Quantitative reverse transcription PCR (qRT-PCR)

Small RNAs of HepG2 cells were isolated using the RNA purification kit following the manufacturer’s instruction (RNAiso for Small RNA, TaKaRa, Dalian, China). The quality of RNA was assayed by calculation of the RNA integrity number (RIN) [27]. High quality of RNA (RIN value was greater than 8.0) was used for reverse transcription of the first-strand cDNA synthesis by reverse transcription using M-MLV reverse transcriptase (TakaRa, Dalian, China). The sequences of the primers used for reverse transcription of mature miRNA with stem-loop structure were listed in Table 2, which were designed according to the corresponding sequence from miRBase database. The quantitative real-time RT-PCR (qRT-PCR) was used for accessing miR-21 expression profile [28,29], which was performed on a Roche lightcycler (LightCycler 480) using TaKaRa SYBR Green I kit (Takara, Dalian, China); the thermal cycling condition for PCR was 95°C for 30 sec, 40 cycles of 95°C for 5 sec, 60°C for 20 sec and 72°C for 20 sec, followed by 40°C for 20 min. The primer sets used for RT-PCR of U6 promoter and miR-21 were listed in Table 2. The control was always included to normalize each reaction with respect to RNA integrity, sample loading and inter-PCR variations. The relative expression ratio was calculated from the real-time PCR efficiencies and the crossing point deviation of experimental samples *vs* controls [30]. The specificity of PCR was determined by sequencing of the PCR products.

**Table 2:** The sequences of primers used for reverse transcription and PCR

**Application**	**Primer**	**Sequence (5’→3’)**
Reverse transcription	miR-21 RT	CTCAACTGGTGTCGTGGAGTCGGCAATTCAGTTGAGTCAACATC
	U6 RT	AACGCTTCACGAATTTGCGT
qRT-PCR of miR-21	Common primer	CTCAACTGGTGTCGTGGA
	miR-21 PCR	ACACTCCAGCTGGCTAGCTTATCAGACTGATG
qRT-PCR of U6	U6 promoter forward	CTCGCTTCGGCAGCACA
	U6 promoter reverse	AACGCTTCACGAATTTGCGT

### Experimental validation of miR-21 target

In order to validate the MAP2K3 mRNA was a target of miR-21, a reporter plasmid containing luciferase with the 3’UTR sequence of MAP2K3 mRNA was generated. The following primers were designed based on GenBank database (NM_ 002756.4), and were used for amplification of wild-type and mutated 3’UTR of MAP2K3 mRNA: the sequence of common forward primer was 5’-GG*ACTAGT*GCGGTTCCCTTACGAGTC-3’, reverse primer for the wild-type of MAP2K3 mRNA 3’UTR was 5’-CG*ACGCGT*CCAAAGCCGGGATAGAGG-3’, and the reverse primer for mutated MAP2K3 mRNA 3’UTR was 5’-CG*ACGCGT*GATCTCAGGTGTGGGTGAGCACTGC-3’; restriction sites of *Spe* I and *Mlu* I were also introduced in the forward and reverse primers, respectively. The cDNA generated from HepG2 RNA was used as templates for amplification of MAP2K3 3’UTR fragment by a PCR assay. The wild-type and mutated 3’UTR fragment were then cloned into the downstream of luciferase reporter gene of pMIR-Report vector (Invitrogen, Grand Island, NY, USA), by which the respective MAP2K3 mRNA luciferase reporter vectors, pMIR-Report/MAP2K3 (harboring wild-type 3’UTR) and pMIR-Report/Mut-MAP2K3 (containing a mutated 3’UTR) were generated. The specificity of miR-21 targeting MAP2K3 mRNA was ascertained by co-transfection plasmid DNA of pAd/pri-miR-21, pAd/miR-21/inhibitor or pAd/con and pMIR-Report/MAP2K3 or pMIR-Report/Mut-MAP2K3 into 293 T cells and determined by the relative activity of firefly luciferase unit (RLU) at 48 h post-transfection using a dual-luciferase Reporter assay kit (Promega, Madison, WI, USA). A Renilla luciferase expressing plasmid pRL-TK (Promega, Madison, WI, USA) was always included in the transfection to normalize the efficiency of each transfection [31].

### Western blotting analysis

Whole cell lystaes (75 μg) were prepared in a lysis buffer (50 mM Tris-HCl, pH 7.5, 5 mM EDTA, 150 mM NaCl, 0.5% NP-40), and were resolved by a 10% sodium dodecyl sulfate (SDS)-polyacrylamide gel (SDS-PAGE), followed by being transferred to a PVDF membrane (Millipore, USA). The membranes were probed with rabbit anti-MAP2K3 antibody and anti-GAPDH antibody (Boster, Wuhan, China) or (1:200, Boster, Wuhan, China) were for the interested protein MAP2K3 and endogenous GAPDH for loading control, respectively. The blots were developed using the enhanced chemiluminescence (ECL) reagent (Amersham Biosciences, Piscataway, NJ, USA) after they were incubated with the appropriate peroxidase labeled secondary antibodies. The protein expression levels were quantified by optical densitometry using ImageJ Software version 1.46 (http://imagej.nih.gov/ij/). Fold change was calculated as the ratio between the net intensity of each sample divided by control GAPDH and the Ad/pri-miR-21, Ad/miR-21/inhibitor and Ad/con infected samples divided by the GAPDH [32].

### MTT assay

Cell proliferation was determined by using the MTT cell proliferation kit (Solarbio, Beijing, China). 5×10^3^ of HepG2 cells were seeded in each 96-well plate and allowed to adhere overnight. The cells were then infected with adenovirus vector at MOI of 10 for the indicated times prior to they were used for MTT assay per the manufacturer’s instruction (Bio-Rad Laboratories, Inc., Irvine, CA, USA).

### Immunohistochemistry staining

The expression of MAP2K3 in clinic human HCC and matched adjacent non-tumor tissues was evaluated by immunohistochemistry staining using rabbit anti-MAP2K3 antibody (1:100, Boster, Wuhan, China). The archival paraffin-embedded sections (5 μm) were deparaffinized and rehydrated through graded alcohol solution. Tissue sections were microwaved in 10 mM sodium citrate pH 6.0 for 13 minutes and cooled down to room temperature (RT) for antigen retrieval. Followed by treating the sections with 0.3% hydrogen peroxide in phosphate buffered saline (PBS) for 15 minutes to inactivate endogenous peroxidase before they were blocked with blocking buffer (5% donkey serum in PBS) for 2 h at RT. The rabbit anti- MAP2K3 antibody was then applied (1:100 in blocking buffer) on the section and incubated overnight at 4°C. Paralleled sections incubated with normal rabbit IgG was used for negative controls. After washing for 3 × 5 min in PBS, sections were incubated with peroxidase labeled donkey anti-rabbit IgG (ZSGB-Bio ORIGENE, Beijing, China) (1:200 in blocking buffer) for 30 minutes at RT. The MAP2K3 signal was developed with 3, 3'-diaminobenzidine (DAB) peroxidase substrate, followed by counterstaining with hematoxylin if it was applicable. The stained sections were examined and photographed on a Nikon Optiphot II microscope equipped with a camera. The expression of MAP2K3 protein was arbitrarily scored from -, + to +++, based on the intensity and number of positive cells, by a single experienced pathologist (Table 1). The non-counterstained sections were also randomly imaged using a 10× objective lens for five fields of each section, and three sections for each sample were evaluated. The obtained images were then for a semi-quantitative analysis of the MAP2K3 expression by measuring the integrated absorbance (IA) using image analysis software Image-Pro Plus 6.0 (IPP6.0, Media Cybernetics, Silver Spring, MD, USA), and the average of the IA values of each sample was used as an index of the expression of MAP2K3 expression (Table 1) [33].

### Statistical analysis

All data collected in this study was obtained from at least three independent experiments for each condition. SPSS15.0 analysis software was used for the statistic analysis. Statistical evaluation of the data was performed by one-way ANOVA and t-test for comparison of differences between the two groups. A value *p*<0.05 set to represent a statistical difference. Data was presented as the mean ± standard deviations (SD).

## Results

### MAP2K3 is downregulated in human hepatocellular carcinoma

**Figure 1:**
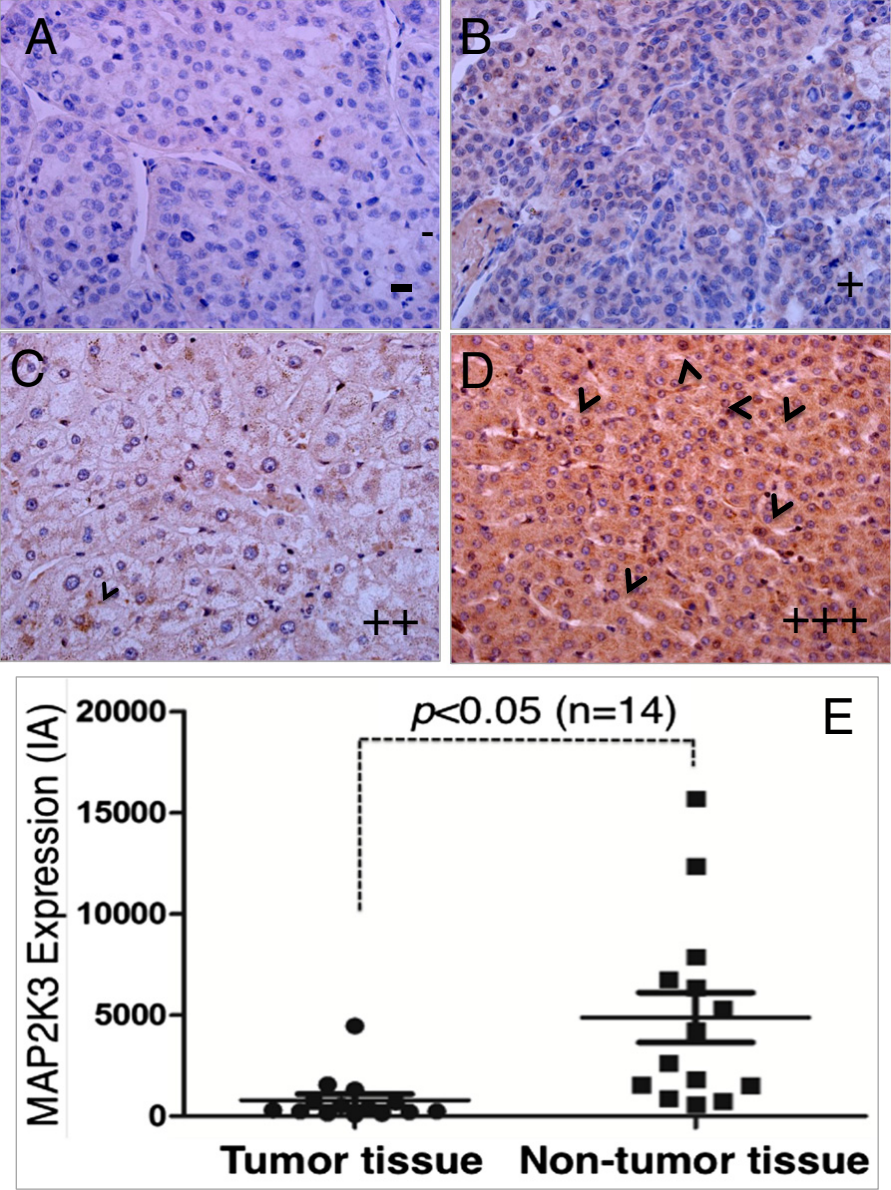
**Immunohistochemistry (IHC) staining determined MAP2K3 expression in human HCC tumor and matched adjacent tissues. A-D**: Representative images of MAP2K3 protein expression determined by IHC staining. **A**: An image represented a negative (-) expression of MAP2K3 expression; **B**: An image represented a low level (+) expression of MAP2K3, which showed a weak immunoreactive staining in cytoplasm; **C**: An image represented a negative (++) expression of MAP2K3 expression; **D**: An image represented a high level (+++) expression of MAP2K3, which exhibited a strong IHC staining in cytoplasm and perinuclear localization (arrowhead). **E**: Semi-quantitative analysis of MAP2K3 protein expression using integrated absorbance (IA) in human HCC tissues. Value was expressed as the average values from each individual sample of HCC tumor tissues or its matched adjacent tissue. The total average value of IA in the HCC tumor tissues was significantly greater as compared with the matched adjacent tissues (*p*<0.05, *n* = 14). Data was expressed as mean ± SD for 14 sets of samples.

To explore clinical relevance of MAP2K3 with the pathogenesis in human HCC, the expression of MAP2K3 was first evaluated in HCC tumor tissues and the adjacent non-tumor tissues by immunohistochemistry staining against anti-MAP2K3 antibody. Immunohistochemistry staining showed predominantly cytoplasmic localization of MAP2K3, with a subset of hematopoietic cells exhibited perinuclear localization for this protein (Figure 1A-1D). Surprisingly, the expression of MAP2K3 was strikingly suppressed in all malignant tumor cells from the fourteen examined archival HCC samples, relative to the adjacent non-tumor tissues, which was supported by a semi-quantitative analysis using an index of the integrated absorbance (IA) for the IHC staining (Figure 1E, Table 1). This result implied that MAP2K3 might be an important signaling molecule that plays a tumor suppressor role in the carcinogenesis of HCC.

### MAP2K3 mRNA is a target of miR-21

**Figure 2:**
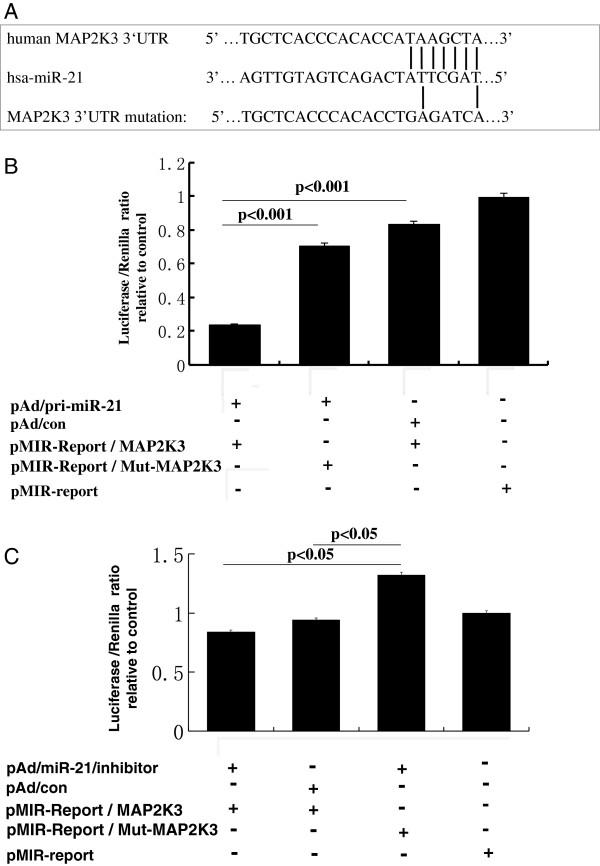
**Validation of MAP2K3 mRNA as a target of miR-21. (A)**: Sequence of potential binding site of miR-21 in the 3’UTR of MAP2K3 mRNA (top panel), mutations were introduced into the binding site for generation of mutated MAP2K3 3’TUR (bottom panel). (**B** and **C**): Validation of miR-21 target using MAP2K3 3’UTR luciferase reporter. Cells co-transfected with pMIR-Report/MAP2K3 3’UTR (WT) or pMIR-Report/Mut-MAP2K3 3’UTR (Mut) and pAd/pri-miR-21 **(B)**, pAd/miR-21/inhibitor **(C)**, and pAd/con plasmids showed a decreased luciferase activity in pAd/pri-miR-21 cells **(B)**. Luciferase activity after site directed mutagenesis of the 3’UTR of MAP2K3 mRNA in the miR-21 seed sequence (pMIR-Report/Mut-MAP2K3) was significantly higher with respect to the pMIR-Report/MAP2K3 vector transfected cells (**B** and **C**). Results represented the mean ± SD from three independent triplicated experiments (N=9).

Since miR-21 has been demonstrated to be elevated in many types of cancer, including the HCC. In order to experimentally validate whether MAP2K3 is a potential target of miR-21 in HCC. Luciferase reporter vector containing a 3’UTR of MAP2K3 mRNA (pMIR-Report/MAP2K3 3’UTR), or a mutated 3’UTR (pMIR-Report/Mut-MAP2K3 3’UTR) were first constructed (Figure 2A). The HepG2 cells were co-transfected with pMIR-Report/MAP2K3 3’UTR or pMIR-Report/Mut-MAP2K3 3’UTR, and proviral plasmid pAd/con, pAd/pri-miR-21 or pAd/miR-21/inhibitor. The results of relative luciferase activity showed a 3.9-fold decrease and an 1.6-fold increase in the cells transfected with pAd/pri-miR-21 and pAd/miR-21/inhibitor, respectively, in comparison with the pAd/con transfected cells (Figure 2B, 2C). There was no significant change of luciferase activity in the cells transfected with pAd/con or pMIR-Report/Mut-MAP2K3 3’UTR plasmid DNA (Figure 2B, 2C). This data indicated that MAP2K3 might be a potential target for oncomir miR-21 in HepG2 cells.

### miR-21 represses MAP2K3 expression in HepG2 cells

**Figure 3:**
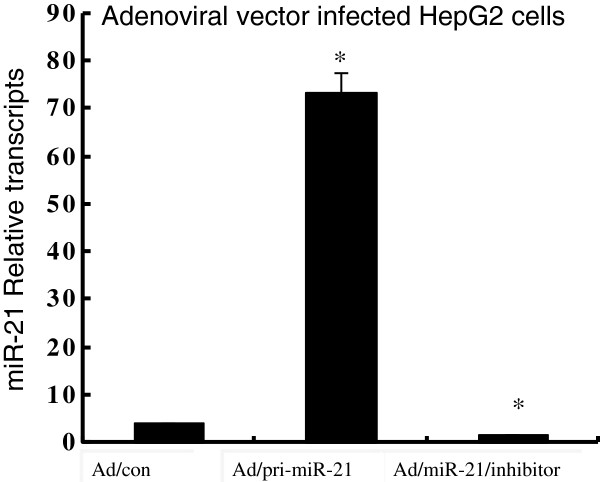
**miR-21 expression in HepG2 cells.** The expression of miR-21 in HepG2 cells infected with Ad/pri-miR-21, Ad/miR-21/inhibitor or Ad/con virus was determined by a qRT-PCR assay. Significantly increased expression of miR-21 was detected in the cells infected with adenovirus Ad/pri-miR-21 (p<0.05); in contrast, a significantly decreased expression of miR-21 was observed in Ad/miR-21/inhibitor infected cells (p<0.05). Compared with pAd/con group, *: *p*<0.05. Results represented the mean ± SD from three independent triplicated experiments (N=9).

**Figure 4:**
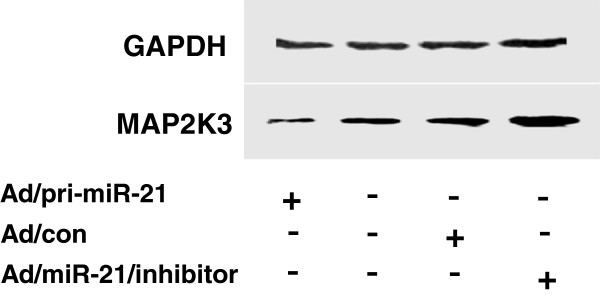
**miR-21 targets MAP2K3 mRNA.** The HepG2 cells were infected with Ad/pri-miR-21, Ad/miR-21/inhibitor or Ad/con adenoviral vector. The expression of MAP2K3 was detected by immunoblotting analysis against anti-MAP2K3 antibody. Compared with Ad/con group, *: *p*<0.05. Data in A represented the mean ± SD from three independent triplicated experiments (N=9).

We next sought to explore whether miR-21 was capable of regulating MAP2K3 in hepatoma cell, HepG2 cells were infected with Ad/pri-miR-21 and Ad/miR-21/inhibitor adenoviral vectors. Although miR-21 has been reported highly expressed in HCC HepG2 cells [13], an 18-fold augmentation and 3-fold inhibition of miR-21 expression were still observed in cells infected with Ad/pri-miR-21 and Ad/miR-21/inhibitor as determined by a qRT-PCR assay [28,29], in comparison with the cells infected with Ad/con, respectively (Figure 3). The total cell lysates were harvested for immunoblotting analysis against anti-MAP2K3 antibody at 24 h post infection. The immunoblotting result demonstrated that the MAP2K3 protein expression was down-regulated by 0.6-fold in cells infected with Ad/pri-miR-21, as compared with the Ad/con (Figure 4); of note, the MAP2K3 expression was augmented by 1.8-fold in cells infected with Ad/miR-21/inhibitor virus (Figure 4). These results suggested that miR-21 was able to down-regulate MAP2K3 expression in HepG2 cells at both of transcriptional post-transcriptional levels, indicative of an underlying mechanism of miR-21 in carcinogenesis of HCC.

### Inhibition of miR-21 expression arrests the proliferation of HepG2 cells

**Figure 5:**
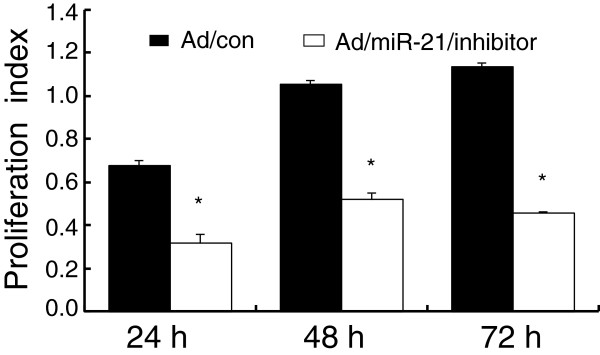
**Impact of miR-21 on HepG2 cell proliferation.** HCC HepG2 cells were infected with Ad/miR-21/inhibitor (miR-21 sponges) or pAd/con virus at MOI 0f 10, the index of cell proliferation was assessed by an MTT assay from 24 to 72 hours. Compared with Ad/con group, *: *p*<0.05. Data in A represented the mean ± SD from three independent triplicated experiments (N=9).

Abundant miR-21 expression was detected in HCC HepG2 cells (Figure 3). In order to better characterize the impact of miR-21 on cancer cell proliferation, the endogenous expression of miR-21 expression was knock down by transduction of miR-21 sponge into HepG2 cells using Ad/miR-21/inhibitor virus. The transduction of miR-21 sponge showed a significant inhibition of cell proliferation in HepG2 cells, in comparison with those transduced with Ad/con, as determined by an MTT assay (Figure 5). This study was consistent with other findings on the contribution of miR-21 as an oncomir in HCC and a potential target for HCC treatment [13,34-36].

## Discussion

Hepatocellular carcinoma (HCC) is one of the most common cancers, which ranks as the third most cancer-related death worldwide [37]. Deregulated expressions of several miRNAs were found correlate with the pathologic and clinical characteristics of HCC [3]. miR-21, one of the most prominent miRNAs involved in the development and progression of many types of cancers, acts as an oncomir in the carcinogenesis of HCC through a mechanism of targeting multiple targets involved in various signaling pathways [34]. miRNA microarray analysis has revealed that miR-21 was dramatically elevated in HCC tumor cells, with significant reductions of the expressions of several tumor suppressor genes, including PTEN, PDCD4, RECK and TPM1 (PTEN) [10-14]. Therefore, identification of novel target of miR-21 will allow us to dissect the underlying signaling pathways regulating liver carcinogenesis, which is crucial for developing novel agents for target therapies of HCC. In the present study, we identified that MAP2K3 was a novel direct target of miR-21. The study of loss-of-function of miR-21 by transduction of miR-21 sponge in HepG2 cells also indicated that miR-21 might regulate cell proliferation, apoptosis and invasiveness partially by targeting MAP2K3 [16,34].

miR-21 has been proposed to contribute to carcinogenesis by targeting several key signaling regulating involved in cell proliferation, apoptosis, invasion and chemoresistance of a variety of cancers [38]. For instance, miR-21 enhanced the cell proliferation by targeting PDCD4 in cervical cancer HeLa cells [39] and gastric cancer [9]. Other instances of miR-21 targeted signaling pathways included TPM1 in breast cancer MCF-7 cells [11], and Matrix Metalloproteinase regulators in glioma cells [14]. The roles of miR-21 in carcinogenesis were also demonstrated by approaches of gain-and/or loss-off-function using transgenic mouse models [40,41]. Using a transgenic mouse model able to conditional overexpressing miR-21 in a tissue-specific manner, in which the expression of miR-21 was under the control of a tissue-specific Nestin promoter, and the transgenic cassette could be conditional knockout by doxycycline-induced Cre-LoxP system, Medina et al. found that over-expression of miR-21 was able to led to a pre-B malignant lymphoid-like phenotype in these mice. In contrast, turning-off transgenic miR-21 expression in the animals led to a complete tumor regression in few days [40]. Similarly, loss-of-function study using a non-small cell lung cancer (NSCLC) transgenic mouse model also suggested that genetic deletion of miR-21 allele was capable of partially protecting the mice from tumor formation [41]. These supportive *in vivo* data strongly suggest that miR-21 is a novel therapeutic target for cancer prevention and treatment.

The emerging of miRNA “sponge” provides a valuable tool for miRNA loss-of function studies in cell lines and transgenic organisms, with several advantages including the a broad range of specificity, applicability and flexibility over chemically modified antisense oligonucleotide inhibitors [23]. A number of studies have demonstrated the application of miRNA sponges with respect to cell type, deliver vector, and type of miRNA targeted, to dominantly negative inhibit the activity of targeted miRNA in cell lines and transgenic organisms [23]. In the this study, an adenoviral vector of miR-21 sponge that containing a tandem of eight binding sites of miR-21 was generated and tested in HCC HepG2 cells, the HepG2 cells transduced with the sponges showed a significant inhibition of miR-21 expression, and as a consequence, the expression of new identified miR-21 targeted gene, MAP2K3 was augmented.

The MAPKs generally expressed in all cell types functionally to transduce extracellular signals into various intracellular responses, and at least four subfamilies of MAPKs have been discovered: extracellular signal-regulated kinase 1 and 2 (ERK1/2), Jun N-terminal kinases (JNKs), P38 MAPKs and ERK5. These distinct MAPK pathways share several common upstream kinases and various downstream targets, suggesting they may crosstalk with one another in various contexts [18]. The mitogen-activated protein kinase-kinase 3 (MAP2K3) belongs to a dual specificity MAPK kinase group (MKK^-^) and is activated by MKK kinase (MKKK) proteins (MEKK1–4) through Ser-189 and Thr-193 phosphorylation. MAP2K3 is an upstream activator of the p38 MAPK protein [42]. Recent studies found that MAP2K3 was down-regulated in immortal human breast epithelial cells and that up-regulation of MAP2K3 expression promoted cell senescence [21]. Zhu et al. found that miR-21 was up-regulated in HCC cells and tissues, which was associated with the capacity of cancer cell migration and invasion in HCC, where the miR-21 expression was inversely correlated with the protein expression of its targeted gene, programmed cell death 4 (PDCD4) and signaling molecules of its downstream pathway [13]. Consistent with this finding, the result present in this study for the first time demonstrated that MAP2K3 was a novel target for miR-21 in HCC, in which MAP2K3 expression was inversely correlated with the miR-21, suggesting an underlying mechanism by which the elevated miR-21 post-transcriptionally down-regulates MAP2K3 signaling in HCC development.

## Conclusions

These results provide a clinical evidence that MAP2K3 may be a tumor repressor gene, and it is a direct target of miR-21 in HCC, indicating an underlying mechanism by which miR-21 is able to directly target MAP2K3 and inhibit its expression during the carcinogenesis of HCC, at both transcriptional and post-translational levels. We also suggest that targeting miR-21-MAP2K3 pathway may be a promising strategy in the prevention and treatment of HCC.

## Competing interests

The authors declare no competing interests.

## Authors’ contributions

GX designed research and analyzed data. YZ performed the experiments. GX and XL analyzed the data and prepared the manuscript. All authors have read and approved the contents of final manuscript.

## Pre-publication history

The pre-publication history for this paper can be accessed here:

http://www.biomedcentral.com/1471-2407/13/469/prepub
